# Protocol for analyzing root halotropism using split-agar system in *Arabidopsis thaliana*

**DOI:** 10.1016/j.xpro.2023.102157

**Published:** 2023-03-13

**Authors:** Bo Yu, Wenna Zheng, Staffan Persson, Yang Zhao

**Affiliations:** 1 Shanghai Center for Plant Stress Biology, CAS Center for Excellence in Molecular Plant Sciences, Chinese Academy of Sciences, Shanghai 200032, China; 2 Department of Plant & Environmental Sciences, University of Copenhagen, 1871 Frederiksberg C, Denmark; 3 Copenhagen Plant Science Center, University of Copenhagen, 1871 Frederiksberg C, Denmark; 4 Joint International Research Laboratory of Metabolic & Developmental Sciences, State Key Laboratory of Hybrid Rice, SJTU-University of Adelaide Joint Centre for Agriculture and Health, School of Life Sciences and Biotechnology, Shanghai Jiao Tong University, Shanghai, China

**Keywords:** Cell Biology, Microscopy, Model Organisms, Plant Sciences, Molecular Biology

## Abstract

Plant roots sense salt gradients in soil to avoid saline environments through halotropism. Here, we present a protocol to study halotropism with an optimized split-agar system that simulates the salt gradient in soil. We describe steps for preparation of the split-agar system, measurement of Na^+^, and observation of root bending. We then detail segmentation of root cells and visualization of microtubules and cellulose synthases. This system is simple to operate and has broader applications, such as hydrotropism and chemotropism.

For complete details on the use and execution of this protocol, please refer to Yu et al. (2022).^1^

## Highlights

• Highly efficient system to study root halotropism
• Step-by-step instructions to prepare the split-agar system
• Monitoring the cytological process during root halotropic response
• Protocol for visualizing Na^+^ with CoroNa Green

## Abstract (editor highlights)

Publisher’s note: Undertaking any experimental protocol requires adherence to local institutional guidelines for laboratory safety and ethics.

## Graphical abstract

**Figure:**
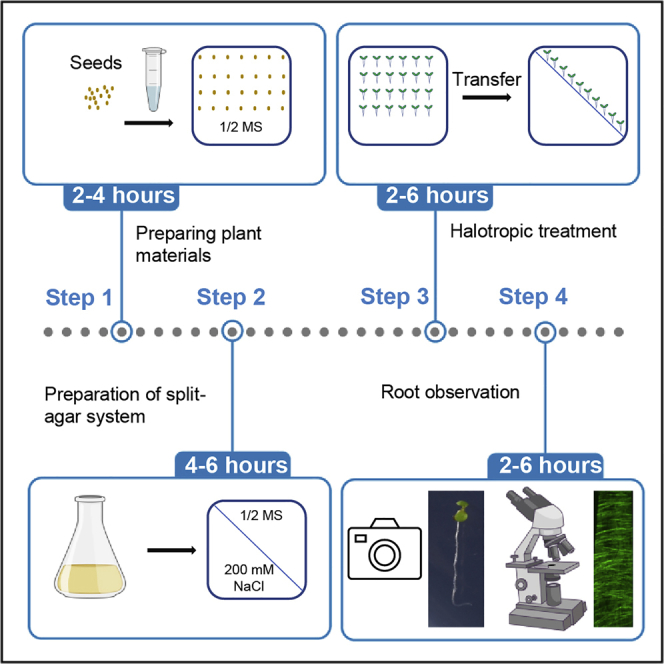

## Before you begin

Halotropism is the tropism of plant roots to avoid saline environments. Based on previous research methods,^2^ we optimized the split-agar system to study root halotropism. In the previous method, seedlings were grown on 1/2 MS medium, and diagonal NaCl gradients were generated by replacing medium after 5 days post-germination without transferring seedlings. Thus, seed germination inconsistency leads to different root lengths, and the distance between root tips and the NaCl/MS interface varies greatly. What we modified is that we selected and transferred the 4–5 day-old seedlings from 1/2 MS medium to the fresh diagonal NaCl gradients plate. Our optimized system can select seedlings with comparable root growth to improve the efficiency of phenotyping analyses and facilitate the comparison between different accessions. In addition, our split-agar system is convenient for controlling the halotropic treatment initiation point and suitable for investigating the effect of exogenous reagents on halotropism, which might be used also for other ions or compounds. The method is designed for Petri dishes, but might be also for other similar containers, such as chambered cover slides (ibidi, 80287).

### Preparation of truncated ruler

**Timing: 2–4 h**

1. Cut a plastic ruler to about 14 cm, the diagonal length, for a 10 cm × 10 cm square disposable Petri dish.
2. Polish the cutting part of the ruler before use.

## Key resources table

**Table:** 

REAGENT or RESOURCE	SOURCE	IDENTIFIER
**Chemicals, peptides, and recombinant proteins**		

Sodium hypochlorite (NaClO)	Sinopharm Chemical Reagent	CAS#:7681-52-9
75% alcohol	General reagent	CAS#: G73537W
Murashige & Skoog (MS) Basal Salt Mixture	Phyto Technology	Cat#: M524
Sucrose	Sinopharm Chemical Reagent	CAS#: 57-50-1
Agar	Sigma-Aldrich	CAS#: 9002-18-0
Ethanol	Sigma-Aldrich	CAS#: 64-17-5
NaCl	Sinopharm Chemical Reagent	CAS#: 7647-14-5
Oryzalin	Sigma-Aldrich	Cat#: 36182
Isoxaben	Sigma-Aldrich	Cat#: 36138
IAA, 3-indoleacetic acid	Sigma-Aldrich	Cat#: I2886
NPA	Sigma-Aldrich	Cat#: 33371
CoroNa™ Green, AM, cell permeant	Thermo Fisher	Cat#: C36676

**Experimental models: Organisms/strains**		

*Arabidopsis thaliana*: Col-0 (4–5 day-old)	Yu et al.^1^	N/A
*Arabidopsis thaliana*: *35S:GFP-TUB6* (4–5 day-old)	Yu et al.^1^	N/A
*Arabidopsis thaliana*: *ABACUS1-80μ* (4–5 day-old)	Jones et al.^3^	CS68847
*Arabidopsis thaliana*: *YFP-NPSN12* (4–5 day-old)	Geldner et al.^4^	N/A
*Arabidopsis thaliana*: *sp2l-4* (4–5 day-old)	Yu et al.^1^	SAIL_117_F12

**Software and algorithms**		

Fiji	Schindelin et al.^5^	https://fiji.sc/
TrackMate 7	Ershov et al.^6^	https://imagej.net/plugins/trackmate/

**Other**		

Percival incubator	Percival	CU36L5
Micropore tape	3M	N/A
Plastic ruler	DELI Co., Ltd (China)	N/A
Square disposable Petri dishes (10 cm × 10 cm)	Haimen Jiebo Co., Ltd (China)	EG1350
Spinning-disk confocal microscope	Nikon	N/A
Chambered coverslip with 2 wells	Ibidi	Cat#80287

## Materials and equipment

**Table:** 

Reagent	Final concentration	Amount
**1/2 MS vertical plates containing 1/2 MS nutrients, 1% sucrose, pH5.7**		

Murashige & Skoog (MS) Basal Salt Mixture	N/A	2.17 g
Sucrose	1%	10 g
Agar	1.2%	12g
ddH_2_O	N/A	Up to 1 L
Total	N/A	1 L

**1/2 MS vertical plates containing 200 mM NaCl, 1% sucrose, pH5.7**		

Murashige & Skoog (MS) Basal Salt Mixture	N/A	2.17 g
Sucrose	1%	10 g
Agar	1.2%	12 g
NaCl	200 mM	11.69 g
ddH_2_O	N/A	Up to 1 L
Total	N/A	1 L

Adjust pH to 5.7 with 1 M KOH, and autoclave at 121°C for at least 20 min. Thereafter, cool down to 65°C (1/2 MS medium) or 45°C (1/2 MS medium containing 200 mM NaCl) and pour into a disposable Petri dish after autoclave. Store solidified medium at 4°C–8°C for up to 3 months.

## Step-by-step method details

Here, we outline the steps for the analysis of Arabidopsis root halotropic response.

### Preparing plant materials

**Timing: 7–8 days**

The purpose of these steps is to prepare Arabidopsis seedlings for halotropic treatment.

1. Sterilize the seeds with 5% (v/v) sodium hypochlorite for 10 min in a 1.5 mL Eppendorf tube.
2. Rinse seeds with sterile water for 5 times.
3. Remove the sterilized water.
4. Resuspend the seeds with 0.05% agarose.
5. Prepare 1/2 MS medium (1.2% agar, 1% sucrose, pH 5.7) with a magnetic bar in 1 L bottle and autoclave at 120°C for 20 min.
6. Cool the medium on a magnetic stirrer to about 45°C.
7. Pour autoclaved medium into 10 cm × 10 cm square disposable Petri dishes (about 40 mL/ plate).
8. Cool down the medium for 15 min and dry for 30–45 min to reduce the water content in the medium and prevent water accumulation during plant growth.
9. Dot the seeds on the plate with a 1 mL pipette.

***Note:*** A plate has 4 rows of seeds, with a spacing of 2 cm between each row. Each row contains about 12–14 seeds spaced about 0.5 cm apart.

10. Seal plates with micropore tape (3M).
11. Wrap the plate with aluminum foil and stratify the seeds at 4°C for 3 days.
12. Grow seedlings vertically in a Percival CU36L5 incubator (or similar) at 21°C–23°C under a 16-h light, 8-h dark photoperiod for 4–5 days.

### Preparation of split-agar system with plastic separator

**Timing: 4–6 h**

These steps aim to prepare the split-agar plates with a plastic separator.

**Figure 1:**
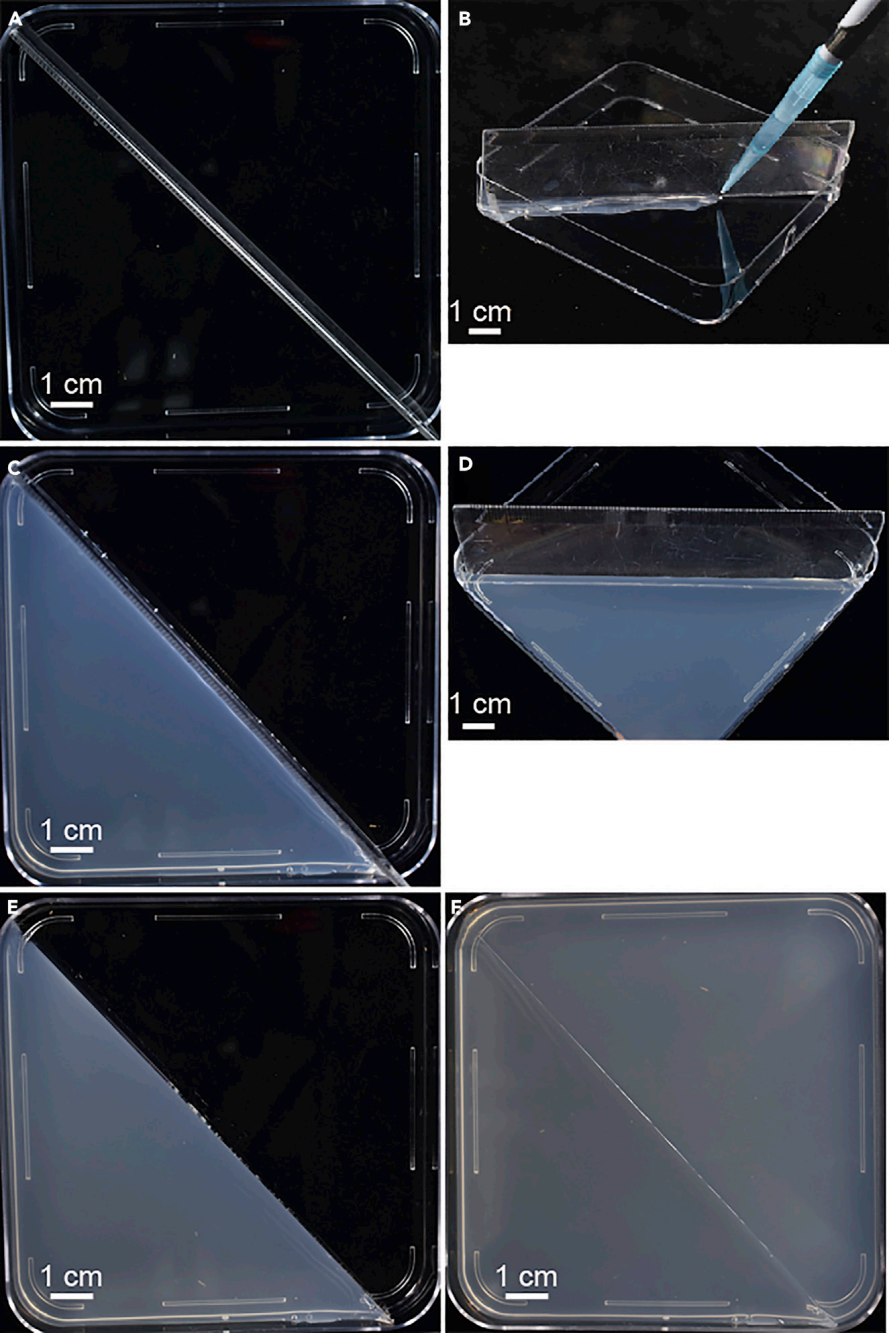
Preparation of split-agar plates with a handmade plastic separator (truncated ruler) (A) Place the ruler vertically along the diagonal of the square disposable Petri dish. (B) Seal the junction with the 1/2 MS medium containing 200 mM NaCl using a pipette. (C) Pour the 1/2 MS medium containing 200 mM NaCl into one side of the square dish and wait until the medium solidifies. (D) Transverse photo of C. (E) Remove the ruler. (F) Pour 1/2 MS medium into the empty side of the Petri dish. Scale bars: 1 cm.

***Note:*** Sterilize the plastic separator with 75% alcohol for 10 min before use.

13. Prepare 1/2 MS medium (1.2% agar, 1% sucrose, pH 5.7) and 1/2 MS medium containing 200 mM NaCl (1.2% agar, 1% sucrose, pH 5.7) with a magnetic bar in 1 L bottle and autoclave at 120°C for 20 min.
14. Cool down the 1/2 MS medium containing 200 mM NaCl on a magnetic stirrer to 45°C. Keep the 1/2 MS medium in a 65°C incubator.
15. Place the handmade plastic separator (truncated ruler, about 14 cm) along the diagonal line of the 10 cm × 10 cm square disposable Petri dish (Figure 1A).

16. Seal the junction of the plastic separator and Petri dish with the 1/2 MS medium containing 200 mM NaCl using a 1 mL pipette (Figure 1B).

***Note:*** The medium flows through the ruler to the other half Petri dish, see the troubleshooting 1.

17. Wait 5 min for the medium to solidify, then pouring the 1/2 MS medium containing 200 mM NaCl into the sealed half Petri dish (Figures 1C and 1D).
18. Wait 20 min for the medium to solidify and remove the plastic separator (Figure 1E).

***Note:*** If it is prone to stick the medium on the ruler, see the troubleshooting 2.

19. Direct pour the 1/2 MS medium kept in the 65°C-incubator into the empty half of the Petri dish (Figure 1F).

***Note:*** In this step, other required reagents or compounds can be added to the 1/2 MS medium.

***Note:*** Temperature may affect the solidification time of agar and the diffusion of Na^+^. Please keep the temperature of 1/2 MS medium as consistent as possible in different batches of experiments.

20. After the medium is solidified (approx. 15 min), and dry it for 30–45 min without the cover.

***Note:*** If water accumulated at the bottom of the square Petri dish, see the troubleshooting 3.

**CRITICAL:** Considering the diffusion of Na^+^. The split-agar plates need to be used within 6 h.

### Preparation of split-agar system by removing the medium

**Timing: 4–6 h**

These steps aim to prepare the split-agar plates by cutting the medium.

**Figure 2:**
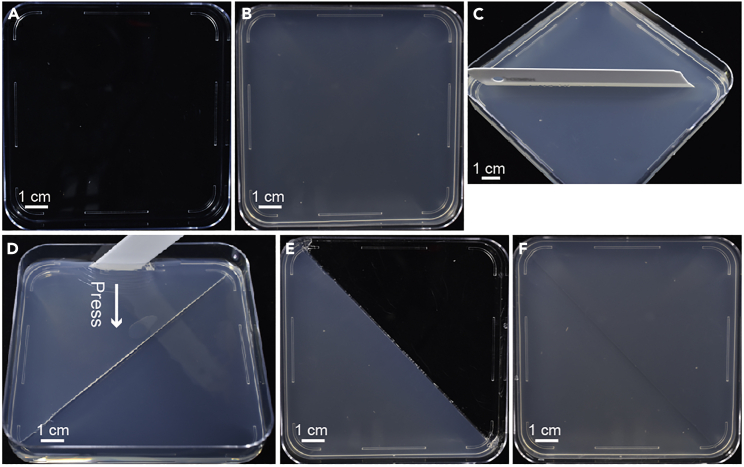
Preparation of split-agar plates by cutting the medium (A) Prepare a square disposable Petri dish. (B) Pour the 1/2 MS medium containing 200 mM NaCl into a square disposable Petri dish and wait until the medium solidifies. (C) Cutting the medium with a blade. (D) Remove the medium by pressing the blade. (E) Cut the medium diagonally and remove half of it. (F) Pour 1/2 MS medium into the empty side of the Petri dish. Scale bars: 1 cm.
24. Wait for 20 min until the medium solidified, and cut the medium diagonally with a blade (Figure 2C).
25. Press the half-medium inward along the interface between the medium and the Petri dish with the blade, and then press the half-medium out along the cut line (Figures 2D and 2E).
26. Pour the 1/2 MS medium that was kept in the 65°C incubator into the empty half of the Petri dish (Figure 2F).

***Note:*** In this step, other required reagents or compounds can be added to the 1/2 MS medium.

***Note:*** Since temperature may affect the solidification time of agar and the diffusion of Na^+^. Please keep the temperature of 1/2 MS medium as consistent as possible in different batches of experiments.

21. Prepare 1/2 MS medium (1.2% agar, 1% sucrose, pH 5.7) and 1/2 MS medium containing 200 mM NaCl (1.2% agar, 1% sucrose, pH 5.7) with a magnetic bar in 1 L bottle and autoclave at 120°C for 20 min.
22. Place the 1/2 MS medium containing 200 mM NaCl on a magnetic stirrer to cool to 45°C. Keep the 1/2 MS medium in a 65°C incubator.
23. Pour the 1/2 MS medium containing 200 mM NaCl into a 10 cm × 10 cm square disposable Petri dish (Figures 2A and 2B).

27. After the medium is solidified for 15 min, dry it for 30–45 min without cover.

***Note:*** If water accumulated at the bottom of the square Petri dish, see the troubleshooting 3.

**CRITICAL:** Considering the diffusion of Na^+^. Split-agar plates need to be used within 6 h.

### Halotropic treatment of Arabidopsis root

**Timing: 2–6 h**

These steps aim to analyze the root halotropism of Arabidopsis seedlings.

**Figure 3:**
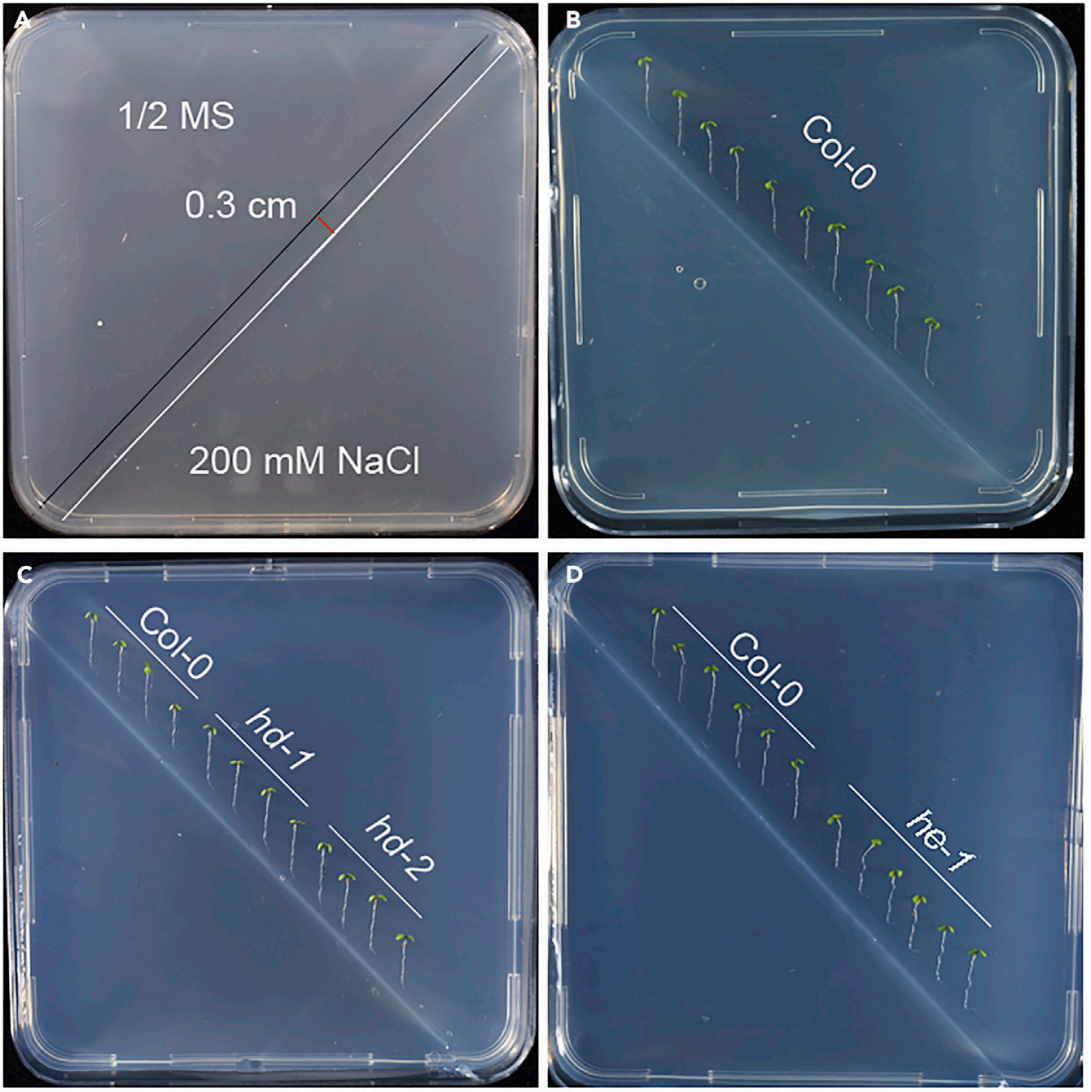
Halotropic treatment of Arabidopsis roots (A) Draw a reference line for root tips on the back of the Petri dish. The white line is the schematic line of the interface of the split mediums. The black line is for the position of the Arabidopsis root drawn with a marker pen. The red line is the distance between the root tips and the interface of the split mediums, about 0.3 cm. (B) The halotropic root growth of wild-type Arabidopsis. (C and D) Halotropic phenotype of halotropic deficient mutants (C) and a halotropic enhanced mutant (D). *hd*: halotropic deficient mutant. *he*: halotropic enhanced mutant.
29. Transfer the 4–5 day-old vertically growth seedlings from 1/2 MS medium to the split-agar plates.
  a. Keep the root tips along the gravity direction
  b. Keep the root tips upright and contact the black line.
  c. Transfer 10–12 seedlings to a Petri dish, with an interval of 0.5–0.75 cm for each seedling (Figure 3B).

***Note:*** If there are different kinds of genotypes, it is better to put them on the same split-agar plate (Figures 3C and 3D).

***Note:*** Reagents or compounds can be added to 1/2 MS medium to test their effects on halotropism (Figures 4A and 4D).

28. On the back of the split-agar plate, find the interface between the 1/2 MS medium containing 200 mM NaCl and 1/2 MS medium (Figure 3A, white line). Draw a straight line on the 1/2 MS medium side 0.3 cm (Figure 3A, red line) parallel with the interface using a marker pen (Figure 3A, black line).

30. Seal the Petri dishes with micropore tape, and put back the treated seedlings into the same culture environment for vertical growth.
31. Visualize the microtubule dynamics after growing for about 1–2 h. Photograph the root growth after 10–16 h of growth.

***Note:*** If the root stops growing after salt avoidance treatment, see the troubleshooting 4.

**Figure 4:**
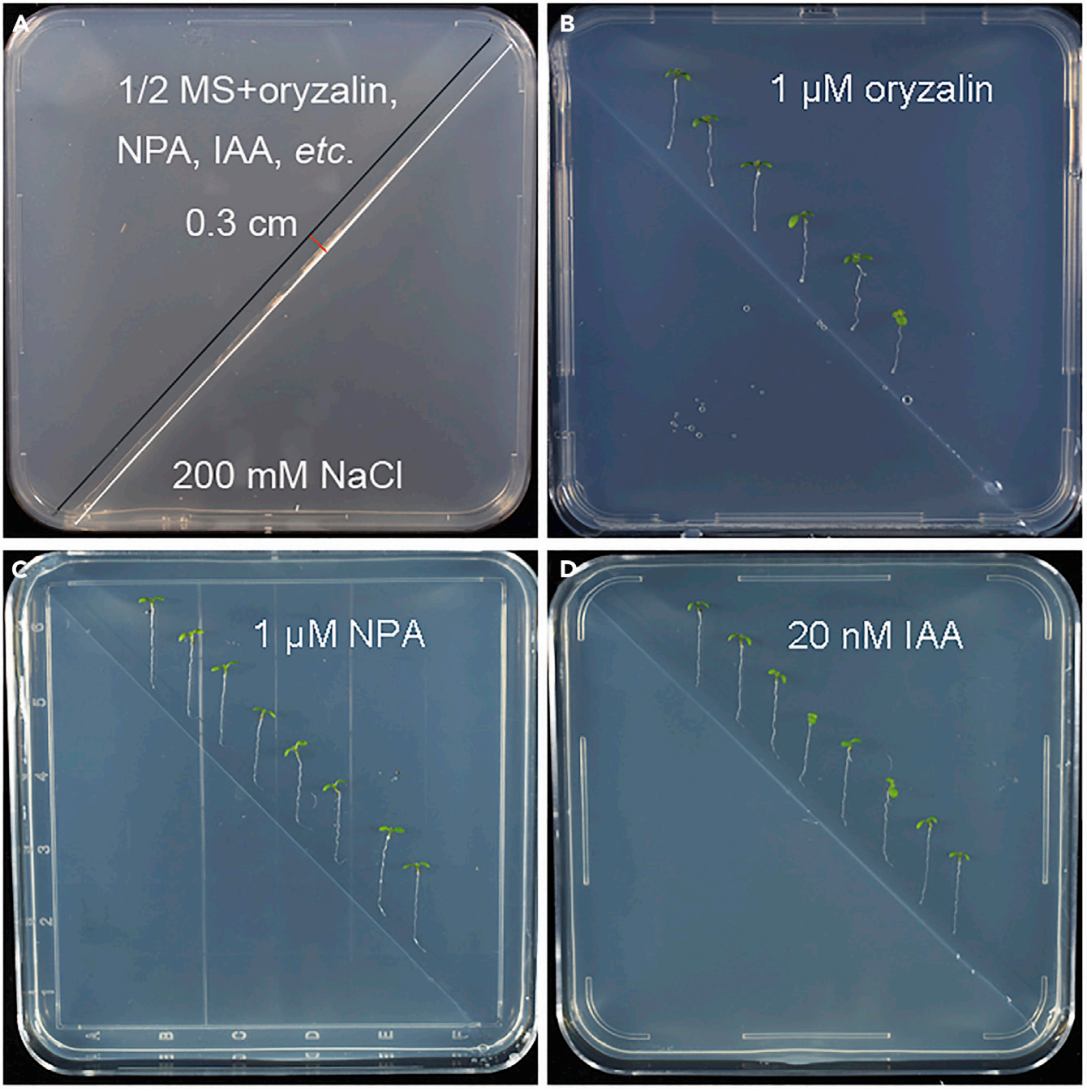
The effects of exogenous reagents on halotropic response (A) Addition of exogenous reagents to 1/2 MS medium. (B–D) Root halotropic growth in the presence of exogenous reagents, including oryzalin (B), N-1-naphthylphthalamic acid (NPA) (C), and indole-3-acetic acid (IAA) (D).

### Visualization of Na^+^ in Arabidopsis root tips

**Timing: 4–8 h**

These steps aim to visualize Na^+^ with CoroNa Green.

32. Prepare 4–5 day-old vertically growing seedlings according to steps 1–12.
33. Transfer the seedlings to a split-agar plate with halostimulation for 3 h according to steps 28–31.
34. Rinse the treated seedlings three times in sterilized water quickly (about 1 s each).

***Note:*** This step is mainly to wash the excess Na^+^ adsorbed on the root surface.

35. Stain the seedlings with 20 μM CoroNa Green (CoroNa Green Sodium Indicator, Thermo Fisher, C36676) for 1 h.
36. Rinse the stained seedlings three times in sterilized water quickly (about 1 s each).

***Note:*** This step is mainly to wash the excess CoroNa Green adsorbed on the root surface.

37. Analyze and image the stained root tips using a confocal microscope. The absorption and emission maxima of the CoroNa Green indicator are at approximately 492 and 516 nm, respectively.

***Note:*** If the fluorescence is too strong after dye application, see the troubleshooting 5.

### Prepare plant materials for observation of root bending under a microscope

**Timing: 6–7 days**

These steps aim to prepare the plant materials.

38. Prepare 20 cm × 20 cm square Petri dishes with 1/2 MS, 1% sucrose and 1.2% agar (adjust pH to 5.7 before autoclave).
39. Sterilize YFP-NPSN12 homozygous seeds with 5% sodium hypochlorite for 10 min in a 1.5 mL Eppendorf tube.
40. Rinse seeds in sterile MilliQ water 5 times.
41. Sow seeds on 1/2 MS medium in a square Petri dish with 0.5 cm intervals.
42. Seal square Petri dish with micropore tape.
43. Stratify the seeds at 4°C for 2 days in the dark.
44. Grow seedlings vertically in an incubator at 23°C for 4 days (16-h light/8-h dark photoperiod).

### Observation of root bending under a microscope

**Timing: 5–6 h**

These steps aim to observe root epidermal cell expansion during root bending.

45. Pour 1/2 MS medium into a chambered cover slide (ibidi, 80287), and make sure to fill the entire chamber by covering it with a sterile glass plate.

***Note:*** For halo-stimulation, replace the bottom left part with 1/2 MS medium containing 200 mM NaCl. For the control treatment, replace the bottom left part with 1/2 MS medium.

46. Install a 20× objective onto the objective inverter (LSM TECH), and turn on the spinning-disk confocal microscope.

***Note:*** The spinning-disk confocal microscopy is an inverted Nikon Ti-E microscope with a CSU-W1 spinning disk head (Yokogawa, Japan), with a deep-cooled evolve charge-coupled iXon Ultra 888 EM-CCD camera (Photometrics Technology, USA).

47. Transfer a 4-day-old seedling with straight root onto the split-agar medium in the chambered cover slide, cover the seedling with a cover slide, and seal it with micropore tape.
48. Place the chambered cover slide vertically on the vertical stage.
49. Adjust to get a clear view of the epidermal cells in the root tip.
50. Start imaging with Z-steps of 0.5 μm intervals covering a depth of more than 10 μm from the root surface to the interior and 10 min time intervals for 3 h.

***Note:*** A 514-nm laser and a 540/50-nm emission filter are used to obtain the YFP signal.

### Segmentation of root epidermal cells

**Timing: 1–2 h**

These steps aim for the segmentation of root cells.

**Figure 5:**
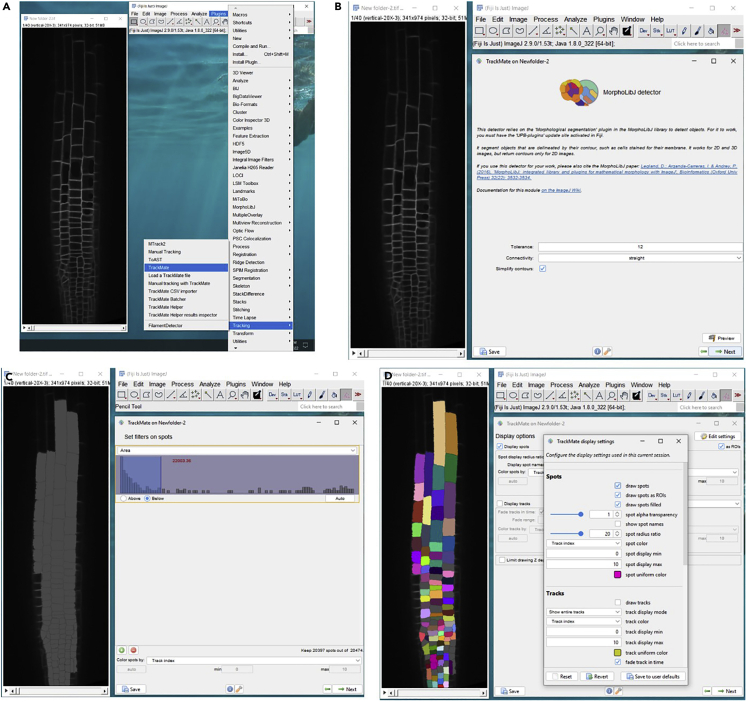
Root cell segmentation (A) Open TrackMate 7 in a new window. (B) Outline the cells. (C) Set filters on spots as an area. (D) Edit settings to get a preferred display.
54. Select a detector: MorphoLibJ detector, and select next.
55. Check “Simplify contours”, select preview, and get a preliminary result with the setting. Adjust to outline most of the cells and select next (Figure 5B).

***Note:*** Use the recommended Tolerance and Connectivity for the first time.

***Note:*** Increase the Tolerance to get less detail.

51. Open Fiji software (make sure it is updated with TrackMate 7) and open the images.
52. Extract the images with clear epidermal cell boundaries at every time point and compose them into a stack (32-bit images).
53. Find Tracking>>TrackMate in the plugins pulldown list and open TrackMate 7 in a new window (Figure 5A).

56. Leave Initial thresholding as it is and select next.
57. Set filters on spots as area and check Below to cover only the cells (Figure 5C).
58. Select Overlap tracker.
59. Edit settings at Display options to get preferred display (set spot alpha transparency to 1) (Figure 5D).
60. At “Select an action”, select Capture overlay, and select execute to export the segmented images.

***Note:*** The finalized images are in one stack, with each cell colored differently. In some cases, the algorithm may not recognize cells as the same cell at different time points, then change the color manually using Flood Fill Tool.

***Note:*** Consult the article “TrackMate 7: integrating state-of-the-art segmentation algorithms into tracking pipelines.” (https://doi.org/10.1038/s41592-022-01507-1) for a more detailed manual.

### Prepare plant seedlings for observation of cortical microtubules and CESAs in root epidermal cells

**Timing: 3–4 days**

These steps aim to prepare the plant seedlings.

61. Prepare chambered cover slides (ibidi, 80287) with about 2 mm-thick 1/2 MS, 1% sucrose, and 1.2% agar (adjust pH to 5.7 before autoclave) in each chamber (Figure 6A).

**Figure 6:**
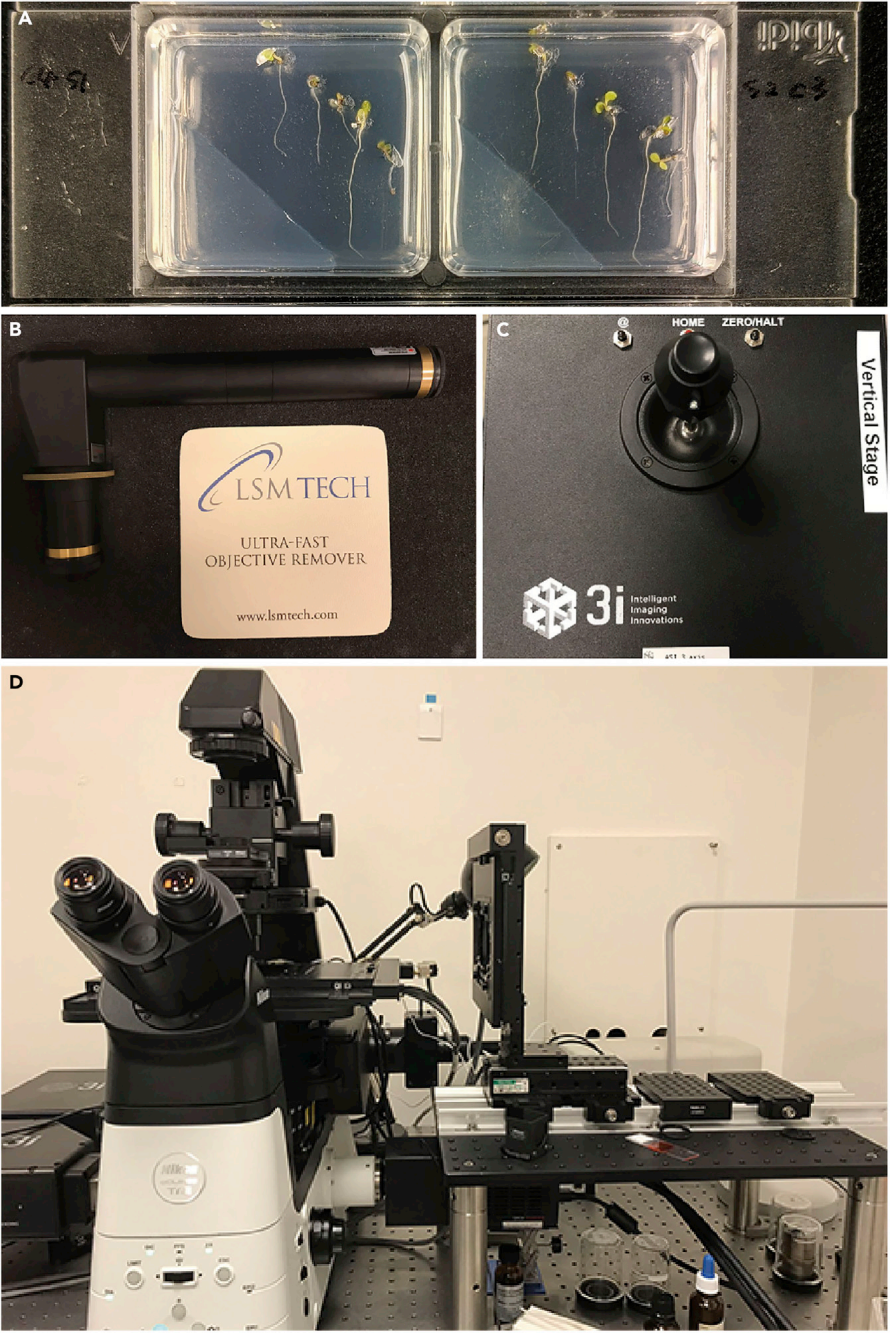
Microscopic detection of Arabidopsis roots in response to halostimulation (A) Prepare Arabidopsis seedlings in a chambered cover slide (ibidi, 80287). (B and C) Confocal vertical stage. (D) The confocal microscope using to observe cytological process in halotropism.
62. Sterilize homozygous transgenic seeds with 5% sodium hypochlorite for 10 min in a 1.5 mL Eppendorf tube.
63. Rinse seeds in sterile MilliQ water 5 times.
64. Sow seeds on the right side of 1/2 MS in chambered cover slides with 0.3 cm intervals, and push the seeds into the medium until they are close to the cover slides in the bottom.
65. Seal chambered cover slides with micropore tape.
66. Stratify the seeds at 4°C for 2 days in the dark.

### Observation of cortical microtubules and CESAs in root epidermal cells

**Timing: 4–5 days**

These steps aim to visualize cortical microtubules and CESAs in root epidermal cells.

67. Grow seedlings vertically in an incubator at 23°C for 4 days (16-h light/8-h dark photoperiod).
68. Halo-stimulation or control treatment of seedlings by replacing the medium.

***Note:*** With halo-stimulation, replace the left corner (about 2 mm away from the longest root tip) of the 1/2 MS medium with 1/2 MS medium containing 200 mM NaCl. For the control treatment, replace the left corner of the 1/2 MS medium with 1/2 MS medium.

69. Install the 100× objective (Apo TIRF, NA 1.49) onto the objective inverter (LSM TECH), and turn on the spinning-disk confocal microscope (Figures 6B–6D).
70. Place a chambered cover slide vertically on the vertical stage.
71. Adjust to get a clear view of the epidermal cells in the root elongation zone.
72. Start imaging with a 0.5 μm interval and 20 μm distance Z-step at a 30 min time interval for 2 h.

***Note:*** Detect GFP signals with a 488-nm laser and a 482/35-nm emission filter, and mCherry signals with a 561-nm laser and a 542/27-nm emission filter, respectively.

**CRITICAL:** Since gravity affects the dynamics of microtubules, a vertical platform is required to avoid gravity interference.

## Expected outcomes

**Figure 7:**
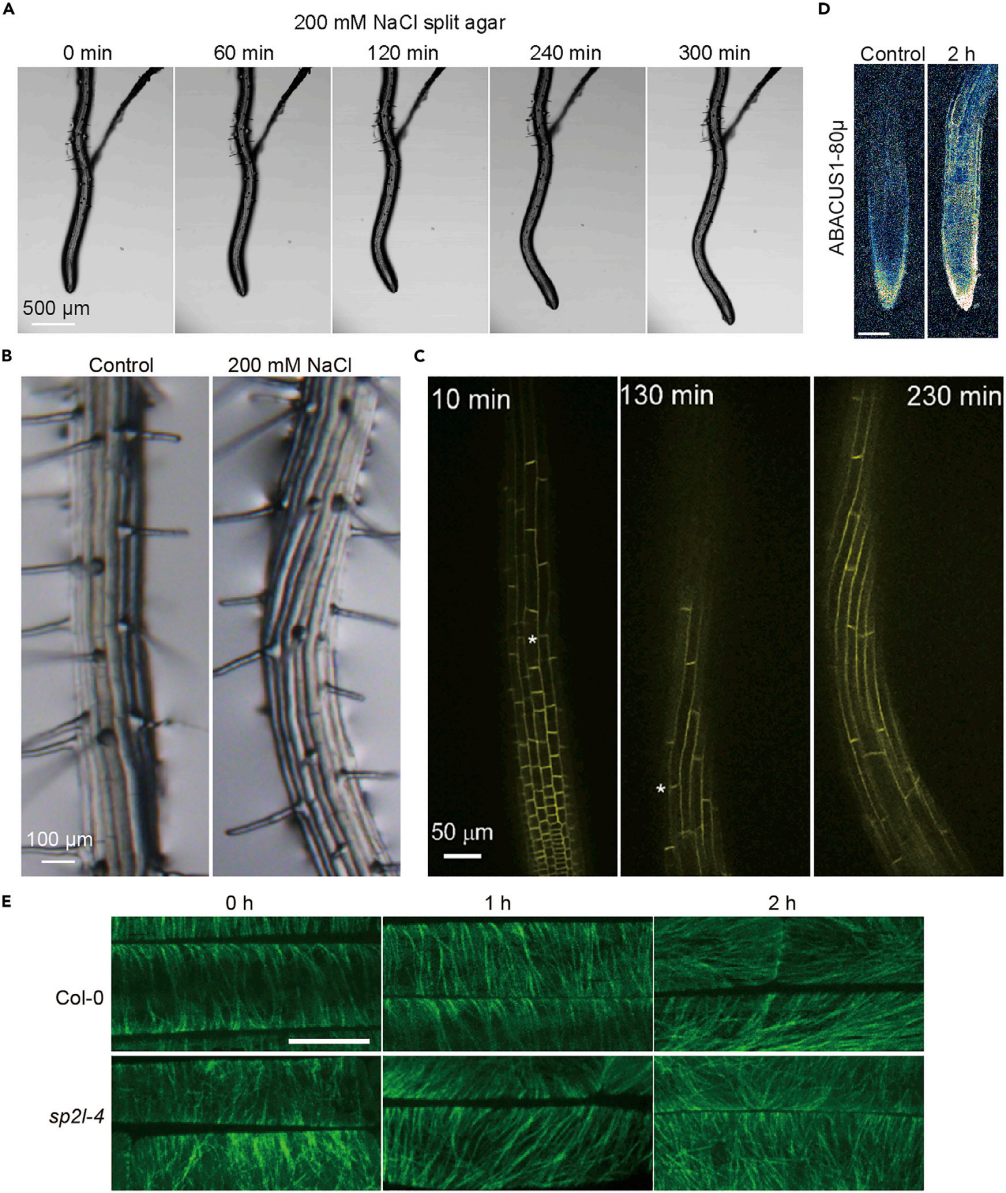
Observation of cytological process during halotropic response (A–C) Root growth was monitored using wild-type seedlings at the corresponding time points under a microscope (A), stereoscope (B), and a Nikon-Andor WD spinning disc confocal with a vertical stage (C). Scale bars: 500 μm in (A), 100 μm in (B) and 50 μm in (C). (D) Monitor the distribution of ABA in root by ABACUS1-80μ without or with halo-stimulation on split-agar medium. Scale bar, 100 μm. (E) Images of cortical microtubules in WT and *sp2l-4* root epidermal cells in the transition zone after halo-stimulation, as visualized by expression of GFP-TUB6. Scale bar, 10 μm. The pictures in Figure 7 refer to our published article, Yu et al.^1^

In the split-agar system, halotropic growth of root tips can be observed in 2–6 h. Therefore, this split-agar system is suitable for screening early salt signaling elements in genetic screens. In addition, we established a split-agar system suitable for microscopy with the chambered cover slides. This is helpful for real-time monitoring of cytological processes during the early halotropic response, such as calcium signals, ROS accumulation, cytoskeleton dynamics, hormone distribution, etc (Figure 7).

## Limitations

Since exogenous reagents need to be added to 1/2 MS at about 65°C, our method is not suitable for reagents or compounds that are thermally unstable.

Importantly, our method cannot completely simulate the soil environment, and other experimental methods are needed to realize the response of the change of root morphology in the soil.

***Note:*** The interface between mediums may form mechanical stimulation.

## Troubleshooting

### Problem 1

The medium flows through the ruler to the other half Petri dish (related to step 16).

### Potential solution

Before pouring the medium, check whether the connection between the ruler and the Petri dish is completely sealed.

### Problem 2

When removing the ruler, it is prone to stick the medium on the ruler, resulting in an uneven section of the medium (related to step 18).

### Potential solution

It may be that the AGAR is not completely solidified, and the solidification time can be increased by 5–10 min in step 18. And do not pull up the ruler when removing it, but quickly push the ruler toward the empty half of the Petri dish.

### Problem 3

Water accumulated at the bottom of the square Petri dish (related to steps 20 and 27). This will make the surface of medium water soaked and affect the attachment of the root, thus affecting root halotropic growth.

### Potential solution

Increase the drying time when preparing the split-agar plates. We suggest that the additional drying time should not exceed 30 min, and the drying time should be consistent for each experiment.

### Problem 4

The root stops growing after salt avoidance treatment (related to step 31).

### Potential solution

The ruler and blade may contain some compounds that have not been removed and affect the root growth. Before using the ruler and blade, wash them with sterile water three times.

### Problem 5

The fluorescence is too strong after dye application (related to step 37).

### Potential solution

Increase the washing times in step 36 and reduce the CoroNa Green concentration (It can be as low as 1 μM).

## Resource availability

### Lead contact

Further information and requests for resources and reagents should be directed to and will be fulfilled by the lead contact, Yang Zhao (yangzhao@psc.ac.cn).

### Materials availability

All the non-commercial materials described in this study are available upon request.

## Data Availability

This study did not generate/analyze datasets.

## References

[1] Yu B., Zheng W., Xing L., Zhu J.K., Persson S., Zhao Y. (2022). Root twisting drives halotropism via stress-induced microtubule reorientation. Dev. Cell.

[2] Galvan-Ampudia C.S., Julkowska M.M., Darwish E., Gandullo J., Korver R.A., Brunoud G., Haring M.A., Munnik T., Vernoux T., Testerink C. (2013). Halotropism is a response of plant roots to avoid a saline environment. Curr. Biol..

[3] Jones A.M., Danielson J.A., Manojkumar S.N., Lanquar V., Grossmann G., Frommer W.B. (2014). Abscisic acid dynamics in roots detected with genetically encoded FRET sensors. Elife.

[4] Geldner N., Dénervaud-Tendon V., Hyman D.L., Mayer U., Stierhof Y.-D., Chory J. (2009). Rapid, combinatorial analysis of membrane compartments in intact plants with a multicolor marker set. Plant J..

[5] Schindelin J., Arganda-Carreras I., Frise E., Kaynig V., Longair M., Pietzsch T., Preibisch S., Rueden C., Saalfeld S., Schmid B. (2012). Fiji: an open-source platform for biological-image analysis. Nat. Methods.

[6] Ershov D., Phan M.-S., Pylvänäinen J.W., Rigaud S.U., Le Blanc L., Charles-Orszag A., Conway J.R.W., Laine R.F., Roy N.H., Bonazzi D. (2022). TrackMate 7: integrating state-of-the-art segmentation algorithms into tracking pipelines. Nat. Methods.

